# Clinical Profile of Patients With Pseudomonas aeruginosa Infection and Antimicrobial Susceptibility Patterns of Pseudomonas aeruginosa: A Cross-sectional Prospective Study From a Tertiary Care Hospital in Central India

**DOI:** 10.7759/cureus.117589

**Published:** 2026-10-07

**Authors:** Salapakshi Nagaraju, Kailash Jatav, Prashanth K Guddeti

**Affiliations:** 1 Department of Microbiology, Index Medical College, Hospital & Research Centre, Indore, IND; 2 Department of Microbiology &amp; Immunology, Windsor University School of Medicine, Cayon, KNA

**Keywords:** antibiogram, antimicrobial resistance, antimicrobial susceptibility test, clinical profile, healthcare-associated infection, icu, intensive care unit, multidrug resistance, pseudomonas aeruginosa, tertiary care hospitals

## Abstract

Background: *Pseudomonas aeruginosa* is an important healthcare-associated opportunistic pathogen in healthcare settings, particularly among critically ill and hospitalized patients. Multidrug resistance, particularly carbapenem resistance, is a major problem that restricts treatment options and increases morbidity rates for patients admitted to intensive care. This study aimed to evaluate the clinical profile of patients with *P. aeruginosa* infection and determine the antimicrobial susceptibility pattern of the isolates in a tertiary care hospital in Central India.

Methods: A hospital-based, cross-sectional prospective study was conducted in the Department of Microbiology, Index Medical College, Hospital and Research Centre, Indore, from January 2023 to December 2025. The study was approved by the Institutional Ethics Committee (ref. no.: MU/Research/EC/Ph.D/2023/028). A total of 171 non-duplicate clinical isolates of *P. aeruginosa* obtained from patients of all age groups were included. Isolates were identified using standard phenotypic methods, and antimicrobial susceptibility testing was performed by the Kirby-Bauer disk diffusion method according to CLSI 2023 guidelines. Clinical and laboratory data were analyzed using IBM SPSS Statistics, version 27.0 (IBM Corp., Armonk, USA), with the chi-square test used to assess associations.

Results: From a total of 171 *P. aeruginosa* isolates, 72 (42.1%) were recovered from respiratory specimens, followed by pus/wound swabs (24.0%), urine (16.4%), and blood (10.5 %). Most isolates were obtained from ICU patients (69.6%), and male patients constituted 65.5% of cases. Hospital-acquired/ventilator-associated pneumonia was the commonest clinical presentation (35.7%). The highest antimicrobial resistance was observed with ceftriaxone (69.0%), ceftazidime (60.2%), and cefepime (57.9%), followed by aztreonam (55.6%), piperacillin-tazobactam (53.8%), and levofloxacin (49.7%). Resistance to imipenem and meropenem was 44.4% and 40.4%, respectively, whereas polymyxin B showed the lowest resistance (2.3%). Significant associations were observed between antimicrobial resistance and ICU stay, prior antibiotic exposure, mechanical ventilation, urinary catheterization, central line/IV catheter use, diabetes mellitus, COPD, chronic kidney disease, and immunocompromised status (p<0.05).

Conclusion: *P. aeruginosa *showed a substantial antimicrobial resistance burden, particularly among ICU patients. Regular local antibiogram surveillance, antimicrobial stewardship, and stringent infection prevention and control measures are essential for optimizing therapy and limiting the emergence of resistant strains.

## Introduction

*Pseudomonas aeruginosa* is an aerobic, non-fermenting, Gram-negative bacillus and a major opportunistic pathogen that causes healthcare-associated infections globally [1]. It commonly causes respiratory tract infections, urinary tract infections, bloodstream infections, wound infections, burn wound infections, and surgical site infections, particularly among critically ill, immunocompromised, and hospitalized patients. Resistance profiles vary considerably among hospitals and geographical regions; hence, continuous surveillance through institution-specific antibiograms is indispensable [2]. It also acquires resistance through chromosomal mutations and horizontal transfer of resistance genes, resulting in multidrug-resistant (MDR) and extensively drug-resistant (XDR) strains [3,4].

The rapid emergence of antimicrobial resistance in *P. aeruginosa* has become a significant global public health challenge [4]. Infections caused by resistant isolates are associated with prolonged hospitalization, increased healthcare costs, treatment failure, and higher mortality rates [4,5]. Recognizing its clinical importance, the World Health Organization has listed carbapenem-resistant *P. aeruginosa* among the critical-priority bacterial pathogens for which new antibiotics and effective therapeutic strategies are urgently needed [6].

Knowledge of local antimicrobial susceptibility patterns is essential for selecting appropriate empirical antibiotic therapy and implementing effective antimicrobial stewardship programs [4]. Recent studies from tertiary care hospitals in India have demonstrated increasing resistance to cephalosporins, fluoroquinolones, and carbapenems, while colistin and certain newer β-lactam/β-lactamase inhibitor combinations continue to retain relatively better activity against most isolates [2,6,7].

Although several studies have evaluated the antimicrobial susceptibility of *P. aeruginosa* in different parts of India, data from Central India correlating the clinical characteristics of infected patients with the antibiogram profile remain limited [2,7]. The present study was undertaken to evaluate the clinical profile of patients with *Pseudomonas aeruginosa* infections and to determine the antimicrobial susceptibility (antibiogram) pattern of the isolates obtained from various clinical specimens in a tertiary care hospital in Central India. The specific objectives were to identify the demographic and clinical characteristics of patients infected with *P. aeruginosa*, to determine the distribution of isolates among different clinical specimens, to analyze the antimicrobial susceptibility pattern of the isolates against commonly used antibiotics, to estimate the prevalence of MDR *P. aeruginosa* isolates, wherever applicable, and to generate local antibiogram data that can guide empirical antimicrobial therapy, support antimicrobial stewardship initiatives, and strengthen infection prevention and control strategies within the hospital.

## Materials and methods

The present cross-sectional study was conducted in the Department of Microbiology, Index Medical College, Hospital & Research Centre (IMCHRC), Indore, from January 2023 to December 2025, after obtaining approval from the Institutional Ethics Committee (ref. no.: MU/Research/EC/Ph.D/2023/028). A total of 171 non-duplicate, consecutive *Pseudomonas aeruginosa* isolates were obtained from patients of all age groups and both sexes attending the outpatient department, inpatient department, and intensive care units of the tertiary care hospital during the study period. Eligible specimens comprised respiratory samples (including sputum, endotracheal aspirates, tracheal aspirates, endotracheal tips, tracheostomy tips, and suction samples), blood, urine, pus, wound swabs, and other sterile body fluids. Patients who provided written informed consent for participation in the study were included. Clinical isolates other than *P. aeruginosa*, contaminated or inadequately collected specimens, and specimens from patients who did not provide informed consent were excluded from the study. The minimum sample size was estimated using the following formula:

$n = \frac{Z_{1-\alpha/2}^2 \cdot p \cdot q}{d^2}$,

where the estimated population proportion (p) = 0.20, q = 1 − p = 0.80, the confidence level (1 − α) was 95% with a corresponding Z-value of 1.96, and the absolute precision (d) was fixed at 0.06, leading to a sample size of 170.74, which was rounded up to 171.

The study included a minimum of 171 clinical isolates of *P. aeruginosa*. Data were collected using a predesigned and validated case record form (CRF) to record the demographic characteristics, clinical profile, hospital location, underlying comorbidities, type of clinical specimen, and relevant treatment details of patients from whom *P. aeruginosa* was isolated. The collected data were entered into IBM SPSS Statistics, version 27.0 (IBM Corp., Armonk, USA) for statistical analysis. Laboratory data were obtained using standard microbiological procedures, including culture and phenotypic identification tests, catalase, oxidase, motility, urease, citrate utilization, oxidative-fermentative glucose test, nitrate reduction, hemolysis, and growth at 42°C. All media and reagents used in the study were subjected to quality-control testing to ensure sterility and satisfactory performance. Quality control for antimicrobial susceptibility testing was performed using *E. coli *ATCC 25922. Antimicrobial susceptibility testing was carried out using the Kirby-Bauer disc diffusion method on Mueller-Hinton agar, and the results were interpreted according to the Clinical and Laboratory Standards Institute (CLSI) M100, 33rd edition (2023) guidelines [8]. The *P. aeruginosa *isolates were tested against the following antimicrobial discs (HiMedia Laboratories, Mumbai, India): piperacillin-tazobactam (PIT, 100/10 μg), ceftazidime (CAZ, 30 μg), cefepime (CP, 30 μg), ceftriaxone (CTR, 30 μg), amikacin (AK, 30 μg), gentamicin (GEN, 10 μg), tobramycin (TOB, 10 μg), aztreonam (AT, 30 μg), levofloxacin (LE, 5 μg), imipenem (IMP, 10 μg), and meropenem (MRP, 10 μg). Polymyxin B (PB, 300 units) susceptibility was determined by the broth microdilution method, and the minimum inhibitory concentration (MIC) was interpreted according to the CLSI M100 guidelines [8].

## Results

A total of 171 non-duplicate *Pseudomonas aeruginosa* isolates were obtained from various clinical samples of patients attending the tertiary care hospital during the study period. The distribution of the 171 *P. aeruginosa* isolates according to clinical samples, ward distribution, age group, and gender is presented in Table 1. Respiratory samples (sputum, endotracheal aspirates, bronchoalveolar lavage, and tracheal aspirates) accounted for the majority of isolates (72; 42.1%), followed by pus/wound swab (41; 24.0%), urine (28; 16.4%), and blood (18; 10.5%). The majority of *P. aeruginosa* isolates (119; 69.6%) were obtained from ICU patients, while 52 (30.4%) were from general wards. Regarding age distribution, the maximum number of isolates were from patients in the age groups 51-60 years (22.2%) and >60 years (22.2%), followed by 41-50 years (18.1%). Male patients (112; 65.5%) were more commonly affected than female patients (59; 34.5%), with a male-to-female ratio of approximately 1.9:1.

**Table 1:** Distribution of Pseudomonas aeruginosa isolates according to clinical samples and demographic characteristics (n = 171) ETA: endotracheal aspirates; BAL: bronchoalveolar lavage

Characteristic	Category	Number of isolates (%)
Clinical samples	Respiratory samples (sputum, ETA, BAL, tracheal aspirates)	72 (42.1%)
	Pus/wound swab	41 (24.0%)
	Urine	28 (16.4%)
	Blood	18 (10.5%)
	Body fluids (pleural, CSF, ascitic)	7 (4.1%)
	Others (ear swab, tissue, catheter tips)	5 (2.9%)
Ward distribution	Intensive care unit (ICU)	119 (69.6%)
	General wards	52 (30.4%)
Age group	≤20 years	11 (6.4%)
	21-30 years	28 (16.4%)
	31-40 years	25 (14.6%)
	41-50 years	31 (18.1%)
	51-60 years	38 (22.2%)
	>60 years	38 (22.2%)
Gender	Male	112 (65.5%)
	Female	59 (34.5%)

The antimicrobial resistance pattern of the 171 *P. aeruginosa* isolates against 11 different antibiotics from various classes is presented in Table 2. The highest resistance rates were observed among cephalosporins, with ceftriaxone showing 69.0% resistance, followed by ceftazidime (60.2%) and cefepime (57.9%). Monobactam aztreonam showed 55.6% resistance, and piperacillin-tazobactam showed 53.8% resistance. Carbapenem resistance was observed as 44.4% for imipenem and 40.4% for meropenem. Among aminoglycosides, gentamicin showed the highest resistance (47.4%), followed by tobramycin (39.8%) and amikacin (36.3%). Levofloxacin demonstrated 49.7% resistance. Notably, polymyxin B showed remarkably low resistance in 4 (2.3%) isolates, while 167 (97.7%) isolates were categorized as intermediate according to CLSI M100 [8].

**Table 2:** Antimicrobial resistance pattern of Pseudomonas aeruginosa isolates (n = 171)

Antibiotic class	Antibiotic	Resistant, n (%)
Penicillins + β-lactamase inhibitors	Piperacillin-tazobactam (PIT)	92 (53.8%)
Cephalosporins (3rd and 4th gen)	Ceftazidime (CAZ)	103 (60.2%)
	Cefepime (CP)	99 (57.9%)
	Ceftriaxone (CTR)	118 (69.0%)
Monobactams	Aztreonam (AT)	95 (55.6%)
Carbapenems	Imipenem (IMP)	76 (44.4%)
	Meropenem (MRP)	69 (40.4%)
Aminoglycosides	Amikacin (AK)	62 (36.3%)
	Gentamicin (GEN)	81 (47.4%)
	Tobramycin (TOB)	68 (39.8%)
Fluoroquinolones	Levofloxacin (LE)	85 (49.7%)
Polymyxins	Polymyxin B (PB)	4 (2.3%)

The association between antibiotic resistance and various clinical parameters of patients from whom *P. aeruginosa* was isolated is presented in Table 3. A significantly higher proportion of resistant isolates were recovered from ICU patients (70.6%) as compared to general ward patients (46.2%), with a p-value of 0.004. Patients with prior antibiotic exposure showed significantly higher resistance (69.4%) compared to those without prior exposure (54.8%) (p = 0.001). The presence of mechanical ventilation (70.1% vs. 56.0%), urinary catheterization (67.7% vs. 60.4%), and central line/IV catheter (70.4% vs. 59.8%) were all significantly associated with higher resistance rates (p < 0.05). Underlying comorbidities such as diabetes mellitus, COPD, chronic kidney disease, and immunocompromised status also showed a statistically significant association with increased antimicrobial resistance in *P. aeruginosa* isolates (p < 0.05 for all parameters).

**Table 3:** Association between antibiotic resistance and clinical profile of patients (n = 171) ^†^Resistant isolates include both resistant and intermediate categories. ^**^Statistically significant (p < 0.05).

Clinical parameter	Category	Total isolates	Resistant isolates, n (%)^†^	χ² value	df	Phi (φ)	p-value
ICU stay	ICU	119	84 (70.6%)	8.147	1	0.218	0.004**
	General ward	52	24 (46.2%)				
Prior antibiotic exposure	Yes	98	68 (69.4%)	10.231	1	0.245	0.001**
	No	73	40 (54.8%)				
Mechanical ventilation	Yes	87	61 (70.1%)	7.294	1	0.207	0.007**
	No	84	47 (56.0%)				
Urinary catheterization	Yes	65	44 (67.7%)	5.176	1	0.174	0.023**
	No	106	64 (60.4%)				
Central line/IV catheter	Yes	54	38 (70.4%)	5.929	1	0.186	0.015**
	No	117	70 (59.8%)				
Diabetes mellitus	Yes	47	34 (72.3%)	4.678	1	0.165	0.031**
	No	124	74 (59.7%)				
COPD	Yes	36	26 (72.2%)	4.236	1	0.157	0.040**
	No	135	82 (60.7%)				
Chronic kidney disease	Yes	21	16 (76.2%)	5.012	1	0.171	0.025**
	No	150	92 (61.3%)				
Immunocompromised status	Yes	29	22 (75.9%)	5.473	1	0.179	0.019**
	No	142	86 (60.6%)				

The distribution of clinical profile and predisposing factors among the 171 patients infected with *Pseudomonas aeruginosa* is presented in Table 4. Hospital-acquired pneumonia/ventilator-associated pneumonia was the most common clinical presentation, observed in 61 patients (35.7%). This was followed by cerebrovascular accident/hemorrhagic shock in 42 patients (24.6%), respiratory failure in 38 patients (22.2%), diabetes mellitus in 47 patients (27.5%), prolonged ICU stay (>7 days) in 52 patients (30.4%), COPD in 36 patients (21.1%), hypertension in 33 patients (19.3%), and septicemia in 28 patients (16.4%). Less common presentations included hematological malignancies/immunocompromised status (17.0%), acute kidney injury/chronic kidney disease (12.3%), burns/trauma (10.5%), and surgical site infections (8.8%).

**Table 4:** Distribution of clinical profile/predisposing factors in patients with Pseudomonas aeruginosa infection (n = 171)

Clinical profile/predisposing factor	Number of patients (%)
Hospital-acquired pneumonia/ventilator-associated pneumonia	61 (35.7%)
Cerebrovascular accident/hemorrhagic shock	42 (24.6%)
Respiratory failure	38 (22.2%)
Septicemia	28 (16.4%)
Diabetes mellitus	47 (27.5%)
Hypertension	33 (19.3%)
Chronic obstructive pulmonary disease (COPD)	36 (21.1%)
Acute kidney injury/chronic kidney disease	21 (12.3%)
Burns/trauma	18 (10.5%)
Hematological malignancies/immunocompromised	29 (17.0%)
Surgical site infections	15 (8.8%)
Prolonged ICU stay (>7 days)	52 (30.4%)

## Discussion

In the present study, respiratory samples were the single largest source of *P. aeruginosa* isolates (42.1%), followed by pus/wound swabs (24.0%) and urine (16.4%), and the majority of isolates (69.6%) originated from ICU patients. This pattern is broadly comparable to the observations of Saeed et al. [9], who reported *P. aeruginosa* recovery from 21.2% of clinical samples at a Lahore tertiary hospital, with the highest isolation rate from the ICU (42.9%), followed by wound swabs (35%) and urine (28%). Similarly, Kumari et al. [4], in a five-year Indian surveillance study, found tracheal aspirate to be the leading source (23.3%), followed by pus/wound swab (22.7%) and urine (20.9%), a distribution pattern consistent with the respiratory-sample predominance seen in our series, although the relative contribution of pus/wound isolates was higher in their cohort. Bindu and Saikumar [10], in a smaller Chennai-based study of 37 isolates, reported pus/wound swab as the predominant source (45.9%), which differs from our respiratory-sample predominance and may reflect differences in the case-mix between a surgical referral unit and a general tertiary hospital. Regarding demographic characteristics, our study demonstrated a male predominance, with males accounting for 65.5% of the cases and a male-to-female ratio of 1.9:1. This finding is consistent with the male predominance reported by Samad et al. (54.93%) [11] and Bindu and Saikumar (54.05%) [10], although the proportion of males in our study was higher, reinforcing the consistently higher susceptibility of male patients to *Pseudomonas aeruginosa*.

The antibiogram in our study demonstrated the highest resistance to ceftriaxone (69.0%), ceftazidime (60.2%), and cefepime (57.9%), whereas resistance to the carbapenems imipenem and meropenem was 44.4% and 40.4%, respectively. Polymyxin B showed the lowest resistance, with only 4 (2.3%) isolates classified as resistant and 167 (97.7%) categorized as intermediate. These findings parallel those of Kumari et al. [4], who documented the highest resistance to levofloxacin (69%) and the lowest to tobramycin (51%) among 2,331 *P. aeruginosa* isolates, indicating a comparably high overall resistance burden despite differences in the specific agents most affected. Gautam et al. [12], in a study of ICU isolates from a tertiary care hospital in New Delhi, reported nearly half of *P. aeruginosa* isolates to be resistant to β-lactams, quinolones, and carbapenems, with the lowest resistance observed to aztreonam (21.82%) and piperacillin-tazobactam (29.89%); all Gram-negative isolates were susceptible to colistin. Although their findings indicate retained activity of a polymyxin agent, direct comparison with our findings should be made cautiously because our study evaluated polymyxin B and predominantly classified isolates as intermediate. Taken together, these comparisons indicate that our isolates had an intermediate resistance profile compared with the lower rates seen for smaller, single-centre Indian series and the much higher rates seen for MDR selected sub-populations, while consistently supporting polymyxin as the most effective last resort. The present study found ICU stay, prior antibiotic exposure, mechanical ventilation, urinary catheterization, central line/IV catheter use, diabetes mellitus, COPD, chronic kidney disease, and immunocompromised status to be significantly associated with antibiotic resistance (p < 0.05 for all parameters). This aligns closely with findings of Salim et al. [13] who, in a multicenter case-control study from Kelantan, Malaysia, reported significant associations between MDR *P. aeruginosa *acquisition and ICU stay (p = 0.011), urinary catheterization (p < 0.001), mechanical ventilation (p = 0.007), and central venous catheter use (p = 0.024), corroborating the invasive-device and ICU-related risk pattern observed in our cohort. These consistent cross-study associations demonstrate that indwelling devices and intensive care exposure are still the major modifiable risk factors for resistant *P. aeruginosa* acquisition regardless of geographic setting.

Hospital-acquired pneumonia/ventilator-associated pneumonia (35.7%), cerebrovascular accident/hemorrhagic shock (24.6%), and prolonged ICU stay (30.4%) were the leading predisposing factors identified in our cohort. Notably, these proportions are numerically close to those reported by Guddeti et al. [14] for *Acinetobacter baumannii* isolates from a comparable tertiary-care Central Indian setting, where aspiration pneumonia/pneumonia (35%), cerebrovascular accident/hemorrhagic shock (30.7%), and mechanical ventilation (58%) were the dominant clinical associations, suggesting that the two leading non-fermenting Gram-negative nosocomial pathogens in this region share a largely overlapping predisposing clinical profile centred on respiratory compromise, neurological insult, and prolonged critical care exposure.

## Conclusions

In our study cohort, *Pseudomonas aeruginosa *demonstrated a substantial antimicrobial resistance burden, particularly among patients admitted to the intensive care unit and those with respiratory infections. High resistance rates were observed against cephalosporins and other commonly used antimicrobial agents. Significant associations between antimicrobial resistance and ICU stay, prior antibiotic exposure, mechanical ventilation, urinary catheterization, central line/IV catheter use, and selected comorbidities were also observed. These findings highlight the importance of regular, institution-specific antibiogram surveillance, judicious antimicrobial use, and stringent infection prevention and control measures to support appropriate antimicrobial therapy and limit the emergence and spread of resistant *P. aeruginosa *strains. However, these findings are specific to the patient cohort and tertiary care hospital setting studied in Central India and should be interpreted within this context.
